# A Novel Directional Element for AC Lines in Systems with Inverter-Based Resources

**DOI:** 10.3390/s26102991

**Published:** 2026-05-09

**Authors:** Kun Qian, Minghao Wen, Xiaoting Xue, Zichang Sun

**Affiliations:** 1 School of Electrical and Electronic Engineering, Huazhong University of Science and Technology, Wuhan 430074, China; 2 Nanjing SAC Power Grid Automation Co., Ltd., Nanjing 211153, China; xtxue_hust@outlook.com; 3 Nanjing Power Supply Branch, State Grid Jiangsu Electric Power Co., Ltd., Nanjing 211102, China; zichangsun@163.com

**Keywords:** control–protection coordination, directional element, fault characteristic, inverter-based resource (IBR)

## Abstract

The fault response of inverter-based resources (IBRs) is strongly influenced by their control strategies, which may significantly change the directional information available to line relays during non-ground faults. As a result, conventional directional elements designed according to the fault characteristics of conventional power systems may exhibit poor adaptability in IBR-connected systems. In particular, zero-sequence directional elements cannot be applied to non-ground faults, whereas negative-sequence-based schemes may be adversely affected by the fault-control behavior of IBRs. To address this problem, this paper proposes a directional element for non-ground faults on AC lines in systems with IBRs. First, the positive-sequence measured impedance at the relay locations is analyzed under typical fault conditions, and the dependence of available directional information on the phase characteristic of the IBR fault current is clarified. Then, a control–protection coordinated method is introduced to regulate the fault-current phase of the IBR during faults so that stable and consistent positive-sequence directional features can be established at both line terminals. On this basis, a unified directional criterion is formulated. Finally, PSCAD/EMTDC simulations are carried out to verify the proposed method, and dynamic model experiments are conducted to validate its engineering feasibility. The results show that the proposed element correctly identifies the fault direction under both three-phase and phase-to-phase fault conditions. Additional tests considering measurement noise and opposite-side grid-strength variation further demonstrate the robustness of the proposed criterion. Compared with conventional directional elements, the proposed method improves the adaptability of non-ground fault-direction identification in IBR-connected AC lines.

## 1. Introduction

With the increasing integration of inverter-based resources, power-system fault behavior is becoming markedly different from that of conventional power systems dominated by synchronous generators [1]. Owing to the effects of control strategies, current-limiting mechanisms, and weak-infeed characteristics, the voltage–current phasor relationship, sequence-component characteristics, and equivalent output behavior of IBRs during faults differ substantially from those of conventional sources. Consequently, AC transmission-line protection principles established on the basis of traditional fault characteristics are facing new adaptability challenges [2,3,4].

Directional elements play an important role in AC line protection by identifying fault direction and ensuring selectivity. Their adaptability in systems with IBRs has attracted increasing attention [5,6]. Accordingly, substantial efforts have been made to investigate the performance of directional protection in IBR-based systems [7,8,9,10,11,12]. Positive-sequence quantities can capture the main electrical characteristics under a wide range of fault conditions and therefore attracted early research interest. References [7,8] showed that, after the integration of IBRs, the phase relationship between the positive-sequence voltage and current at the relay location may differ markedly from that in conventional systems dominated by synchronous generators. This deviation is mainly caused by controlled fault-current injection, changes in equivalent impedance characteristics, and weak infeed effects. As a result, the sensitivity of directional elements based on positive-sequence quantities or positive-sequence fault components may be reduced, and in severe cases, a failure to operate may occur. This deterioration becomes more pronounced under severe close-in faults and low-voltage conditions. Negative-sequence quantities are well-suited to characterizing phase faults. Conventional negative-sequence directional elements have therefore been widely used for phase-fault direction identification. References [9,10] reported that IBRs usually adopt negative-sequence current suppression control. As a result, the adaptability of conventional negative-sequence directional protection is degraded, which may lead to both maloperation and failure to operate. Zero-sequence directional protection is widely used for ground-fault protection of high-voltage lines because of its high reliability. References [11,12] showed that, in systems with IBRs, the performance of existing zero-sequence directional protection is largely unaffected. However, it is not applicable to non-ground faults. In summary, existing directional elements still exhibit clear adaptability limitations in systems with IBRs. Zero-sequence directional elements cannot be used for phase-fault identification, negative-sequence directional elements are adversely affected by control strategies, and positive-sequence directional elements may suffer from maloperation, failure to operate, and voltage dead-zone issues. Therefore, fault-direction identification for non-ground faults in IBR-based systems remains an urgent problem.

In recent years, the adaptability of directional protection in systems with IBRs has received increasing attention. To address this issue, several studies have improved directional elements by making use of the fault characteristics of systems with IBRs. In [13], a directional criterion for lines connected to photovoltaic power stations was developed based on the phase relationship between the positive-sequence voltage and line current. In [9], the mechanism responsible for the maloperation of conventional negative-sequence directional elements in inverter-interfaced renewable systems was analyzed, and an adaptive improvement method was proposed. In general, these studies improve the performance of existing directional elements or develop new principles by exploiting the differences between the fault characteristics of IBRs and those of the opposite-side AC system. However, such methods often require different criteria or settings at the two line terminals depending on the source characteristics behind the relays. As a result, their applicability may be reduced when the network topology or the IBR integration pattern changes, and in some cases, correct operation may no longer be guaranteed.

Fault characteristics form the basis for the design of relay protection schemes. In systems with IBRs, increasing attention has been paid to the idea of control and protection coordination, in which the identification requirements of protection are first analyzed, and the control strategies of IBRs are then modified to actively shape fault characteristics favorable for protection operation [14,15]. Following this idea, this paper proposes a directional protection method for non-ground faults in AC systems with IBRs, while taking the safe operating constraints of IBR equipment into account. First, for non-ground fault scenarios, the relationship between the positive-sequence fault quantities at the relay locations on both line terminals and the short-circuit current characteristics of IBRs is analyzed, and the influence of IBR control on the directional information available at the relays is clarified. On this basis, the control strategy of IBRs is improved according to the fault-information requirements of directional protection, so that the fault response can be actively regulated to establish consistent positive-sequence directional features at both line terminals. A unified directional criterion and corresponding setting method are then developed, allowing the same criterion and settings to be applied at both line terminals. Finally, PSCAD/EMTDC simulations are carried out to verify the proposed method, and dynamic-model experiments are conducted to validate its engineering feasibility. The results demonstrate that the proposed method can effectively improve the reliability and practical applicability of fault-direction identification for non-ground faults in systems with IBRs.

Motivated by the above analysis, the main advantages and novelty of the proposed method are summarized as follows.

(1) The concept of positive-sequence measured impedance is introduced to characterize the relay-side voltage–current phase relationship. It provides a simple and clear analytical variable for describing the directional feature of positive-sequence quantities.
(2) An improved IBR control strategy is proposed to actively shape the positive-sequence measured impedance characteristic. Instead of passively relying on the natural fault response of IBRs, the proposed method makes use of the fast controllability of IBRs to regulate the positive-sequence current phase. As a result, the measured impedance characteristics at both line terminals become consistent under non-ground faults, while the characteristics under forward and reverse faults remain clearly distinguishable.
(3) The proposed directional protection principle is simple and has good engineering applicability. The same angular criterion and setting can be used at both line terminals, without modifying the criterion according to the IBR integration position or the source characteristic behind each relay. This reduces the dependence on terminal-specific settings and improves the adaptability of non-ground fault-direction identification in IBR-connected AC lines.

The rest of this paper is organized as follows. Section 2 analyzes the phase relationship between the positive-sequence voltage and current at the relay location. Section 3 presents the proposed directional protection method based on control and protection coordination. Section 4 verifies the proposed method by means of simulations and dynamic-model experiments. Section 5 concludes the paper.

## 2. Characteristics Analysis of Positive-Sequence Voltages and Currents

**Figure 1:**
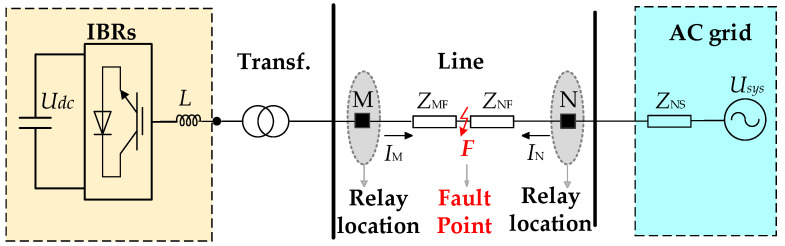
Simplified diagram of a system with IBRs.

For non-ground faults in systems with IBRs, conventional negative-sequence directional protection may fail to operate reliably because of the negative-sequence suppression control usually adopted by IBRs. Since positive-sequence quantities can capture the main electrical characteristics of the system under non-ground faults, it is necessary to further investigate the positive-sequence voltage and current characteristics at the relay location, as well as their dependence on the control strategy of IBRs. The following analysis is carried out on the basis of the typical system with IBRs shown in Figure 1. For phase-to-phase faults, IBRs usually employ negative-sequence suppression control, and the corresponding negative-sequence network can be approximately treated as open-circuited.

### 2.1. Three-Phase Fault

**Figure 2:**
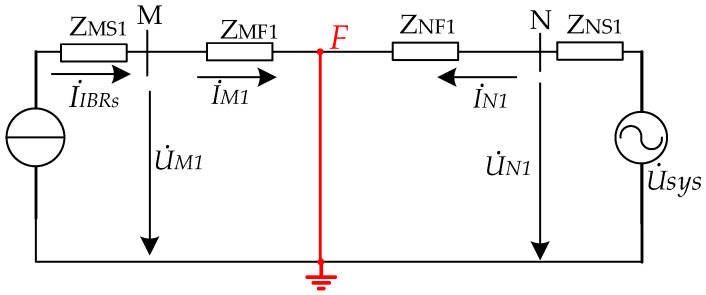
Equivalent circuit of power grid with IBRs under three-phase fault.

Under a three-phase short-circuit fault on an AC line, the system contains only positive-sequence components. It is therefore necessary to analyze the phase relationship between the positive-sequence voltage and current at the relay location under this condition, so as to provide a basis for the development of the directional element. For non-close-in faults, the positive-sequence voltage at the relay location usually remains at a certain level and can be directly used for phase analysis. By contrast, when the fault is close to the relay location, the measured positive-sequence voltage drops significantly and may approach zero in extreme cases. Under such conditions, its phase information can no longer be reliably identified, giving rise to the voltage dead-zone problem. Since memory quantities can retain the pre-fault phase characteristics after fault inception, they have been widely used in engineering practice for low-voltage-related elements. On this basis, for close-in three-phase faults, it is necessary to further examine the feasibility of constructing a directional criterion based on the phase relationship between the memorized positive-sequence voltage at the relay location and the fault current, thereby providing a basis for addressing the voltage dead-zone problem of the directional element under close-in low-voltage conditions. For the three-phase fault on the line shown in Figure 1, the corresponding sequence network is shown in Figure 2.

For non-close-in faults, sequence-network analysis shows that the phase relationship between the positive-sequence voltage and current at relay locations M and N can be expressed as follows. To establish a unified analytical basis, this paper introduces the positive-sequence measured impedance, defined as the ratio of the positive-sequence voltage to the positive-sequence current at the relay location.

(1) $$\arg Z_{M1}=\arg\frac{{\dot{U}}_{M1}}{{\dot{I}}_{M1}}=\arg(Z_{MF1})$$

(2) $$\arg Z_{N1}=\arg\frac{{\dot{U}}_{N1}}{{\dot{I}}_{N1}}=\arg(Z_{NF1})$$

where the subscripts 1 and 2 denote the positive- and negative-sequence components, respectively. In (1) and (2), ${\dot{U}}_{N1}$, ${\dot{U}}_{M1}$, ${\dot{I}}_{N1}$, and ${\dot{I}}_{M1}$ denote the voltages and currents at the relay locations on the two sides, respectively, whereas $Z_{NF1}$ and $Z_{MF1}$ denote the equivalent line impedances from the relay locations on both sides to the fault point.

To establish a unified analytical basis, this paper introduces the positive-sequence measured impedance, defined as the ratio of the positive-sequence voltage to the positive-sequence current at the relay location.

Under a three-phase fault, the phase difference between the positive-sequence voltage and current at the relay location is determined by the impedance angle of the equivalent impedance between the fault point and the relay location. In particular, when a solid three-phase short circuit occurs close to the relay location, the terminal voltage may drop to nearly zero, making it difficult to accurately measure the positive-sequence voltage. Memorized voltage has been widely used in low-voltage-related protection elements to maintain reliable operating performance. Therefore, the phase relationship between the memorized voltage and the current at the relay location can be used to address the voltage dead-zone problem.

For a close-in three-phase fault on the AC line, the measured positive-sequence voltage at the relay location may become too low to provide a reliable phase reference. Therefore, the memorized positive-sequence voltage is used to construct the positive-sequence measured impedance. In this case, the phase angle of the positive-sequence measured impedance, i.e., the measured positive-sequence measured impedance at the relay locations on both line terminals can be expressed as:

(3) $$\arg Z_{M1}^{\mathrm{mea}}=\arg\frac{{\dot{U}}_{\mathrm{sys}}+{\dot{I}}_{IBRs(0)}(Z_{NS1}+Z_{NF1}+Z_{MF1})}{{\dot{I}}_{IBRs}}$$

(4) $$\arg Z_{N1}^{\mathrm{mea}}=\arg\frac{\left({\dot{U}}_{\mathrm{sys}}+{\dot{I}}_{IBRs(0)}Z_{NS1})(Z_{NS1}+Z_{NF1}\right)}{{\dot{U}}_{\mathrm{sys}}}$$

where the subscript *m* denotes the memorized quantity. *Z*_NS1_ is the positive-sequence equivalent internal impedance of the AC grid. *Z*_MS1_ denotes the positive-sequence equivalent internal impedances of the IBRs. ${\dot{U}}_{\mathrm{sys}}$ represents the equivalent AC voltage. ${\dot{I}}_{IBRs}$ denotes the current supplied by the IBR. *I*_IBRs(0)_ denotes the current supplied by the pre-fault IBR. $Z_{M1}^{\mathrm{mea}}$, $Z_{N1}^{\mathrm{mea}}$ donate the positive-sequence measured impedance.

The approximation from (3) and (4) to (5) and (6) follows from the weak-infeed fault characteristics of converter-interfaced resources. Since the IBR fault current is constrained by current-limiting and fault-ride-through controls, it is usually close to the rated current. For converter-interfaced wind power plants, the pre-fault current also depends on the wind power output and may be below the rated value. Thus, the voltage-drop terms associated with the IBR-side fault and pre-fault currents are relatively small compared with the dominant AC-system source and grid-impedance terms. This approximation does not assume zero IBR current, but extracts the dominant phase relationship required for directional discrimination. Accordingly, the phase relationships can be approximately written as:

(5) $$\arg Z_{M1}^{\mathrm{mea}}\approx \arg\frac{{\dot{U}}_{\mathrm{sys}}}{{\dot{I}}_{IBRs}}$$

(6) $$\arg Z_{N1}^{\mathrm{mea}}\approx \arg(Z_{NS}+Z_{NF})$$

Define the phase difference between ${\dot{U}}_{\mathrm{sys}}$ and ${\dot{I}}_{IBRs}$ as $\theta_{UI}$. It follows from (1), (2), (5), and (6) that, when $\theta_{UI}$ equals the system impedance angle, the phase difference between the positive-sequence voltage and current at the relay location is equal to the impedance angle under a forward three-phase fault.

### 2.2. Phase-to-Phase Fault

**Figure 3:**
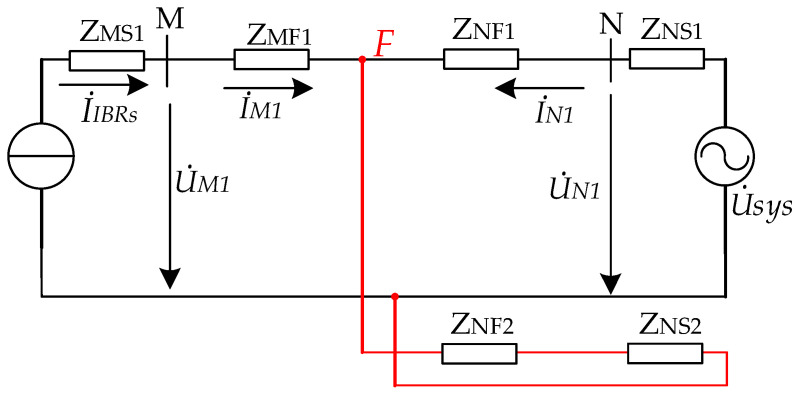
Equivalent circuit of power grid with IBRs under phase-to-phase fault.

Taking the typical system with IBRs shown in Figure 1 as an example, a phase-to-phase fault on the protected line is considered, and the corresponding composite sequence network is shown in Figure 3.

Accordingly, the phase relationship between the positive-sequence voltage and current at relay locations M and N can be written as:

(7) $$\arg Z_{M1}^{\mathrm{mea}}=\arg(\frac{2Z_{MF1}+Z_{N1}}{2}+\frac{1}{2}\frac{{\dot{U}}_{sys}}{{\dot{I}}_{IBRs}})$$

(8) $$\arg Z_{N1}^{\mathrm{mea}}=\arg\frac{(Z_{N1}+Z_{NF1})\frac{{\dot{U}}_{sys}}{{\dot{I}}_{IBRs}}+Z_{NS1}Z_{F2}}{\frac{{\dot{U}}_{sys}}{{\dot{I}}_{IBRs}}-Z_{N1}}$$

Due to the weak-infeed fault characteristics of the IBR, the first terms in the numerator and denominator on the right-hand side of (8) dominate the corresponding second terms. Hence, (8) can be approximated by:

(9) $$\arg Z_{N1}^{\mathrm{mea}}\approx \arg(Z_{N1}+Z_{NF1})$$

For (7), the expression on the right-hand side consists of two terms. The angle associated with the first term is the system impedance angle, whereas that associated with the second term is $\theta_{UI}$. Therefore, the phase difference between the positive-sequence voltage and current lies between these two angles. In conjunction with the analysis of (9), it can be seen that, when $\theta_{UI}$ is equal to the system impedance angle, the phase difference between the positive-sequence voltage and current at both line terminals is equal to the impedance angle under a forward fault.

The above analysis shows that, for a phase-to-phase fault on an AC line in a system with IBRs, the phase relationship between the positive-sequence voltage and current at the relay locations on both line terminals depends on the phase difference between the equivalent system source and the current injected by the IBR. When this phase difference equals the system impedance angle, the phase difference between the positive-sequence voltage and current at the relay locations becomes a constant under a forward fault. By analogy, for a reverse fault, the corresponding angle is equal to the negative system impedance angle. Accordingly, a unified directional criterion can be established from the phase difference between the positive-sequence voltage and current at the relay location for fault-direction identification.

## 3. Proposed Directional Element

The above analysis shows that when the phase lag of the short-circuit current injected by the IBR with respect to the equivalent AC system source equals the system impedance angle, the phase difference between the positive-sequence voltage and current at the relay location exhibits a clear directional feature and can therefore be used for fault-direction identification. In practical AC transmission systems, the impedance angle of overhead lines is generally within 70°~85°, and may approach 90° when the equivalent impedance is highly inductive. Therefore, 90° is adopted as a simplified reference angle for theoretical analysis and IBR fault-current phase control. This setting simplifies the IBR control strategy and avoids adjusting the control target according to the exact impedance angle of each line. Meanwhile, the phase angle of the equivalent AC system source cannot be directly obtained from the electrical quantities available at the relay location, whereas under normal operating conditions, the voltage phase angle at relay location M is close to that of the equivalent source [6]. Accordingly, the control strategy of the IBR is modified to actively regulate the fault-current phase angle, based on which a directional protection method is developed.

### 3.1. Control Strategy Based on Control and Protection Coordination

When the point-of-connection voltage drops below a prescribed threshold, the phase-locked loop (PLL), following existing fault ride-through strategies, is switched from the real-time voltage to the memorized pre-fault voltage phase so as to maintain phase-tracking capability. On this basis, the memorized voltage technique is adopted in this paper to reconstruct the reference voltage during faults, such that the PLL reference frame of the IBR is aligned with the memorized positive-sequence voltage vector at the terminal, and the IBR is controlled to inject a positive-sequence current lagging the reference-point voltage by 90°. Meanwhile, owing to the limited short-circuit overcurrent capability of power electronic devices, a current-limiting strategy must be coordinated during faults to ensure safe operation of the converter.

**Figure 4:**
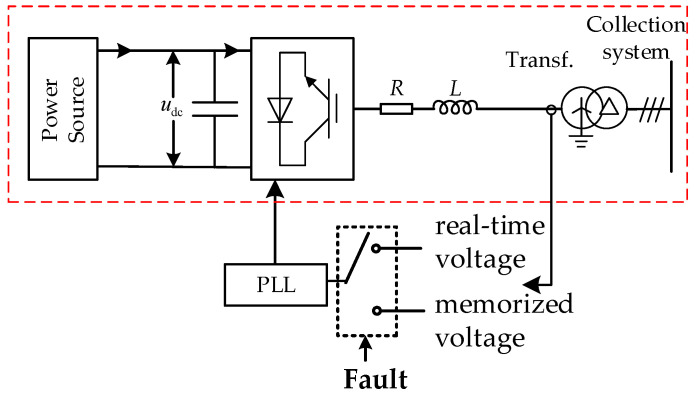
Phase-holding strategy for IBR fault-current phase control.

Figure 4 illustrates the phase-holding strategy employed in the proposed control scheme for regulating the IBR fault-current phase. When a fault causes the PCC voltage to fall below a predefined threshold, the PLL angle is no longer updated by the distorted low-voltage signal. Instead, the pre-fault positive-sequence voltage phase is retained as the reference phase, ensuring that the IBR’s fault-current phase remains consistent during the fault. This phase-holding strategy allows the directional protection to maintain a stable and distinguishable voltage-current phase relationship, which is crucial for accurate fault-direction identification. The diagram demonstrates how the control–protection coordination between the PLL and memorized voltage works to improve fault detection reliability in IBR-connected systems.

The fault control strategy of the IBR should simultaneously satisfy the AC-side overcurrent constraint and the DC-side safety constraint. Because power electronic devices have limited short-term overcurrent capability, an IBR can typically provide only 1.2~2.0 p.u. of rated current during faults. Its output current therefore needs to be constrained within the permissible range by a current-limiting loop. At the same time, the control strategy should also account for the modulation margin of the converter and the safe operation of the DC link, so as to avoid overmodulation caused by an excessive reference voltage or a modulation index approaching its limit, while retaining an adequate safety margin for stable operation. Accordingly, the fault control of the IBR should comprehensively consider current limitations, modulation-index constraints, and DC-side safety requirements, so as to achieve the intended control objective without compromising equipment security. Under these constraints, the current limit $I_{\max1}$ and $I_{\max2}$ are determined as follows:

(10) $$I_{\max1}=k_{\max}I_{nor}$$

(11) $$\left\{\begin{array}{l} \sqrt{V_{1}^{2}+V_{2}^{2}+2V_{1}V_{2}\cos\varphi_{A,B,C}}=0.5m_{dc}U_{dc}\\ V_{1}=(u_{gd1}+I_{\max2}\omega L)+ju_{gq1} \end{array}\right.$$

where V_1_ and V_2_ denote the positive- and negative-sequence components of the reference voltage. $k_{\max}$ is the overcurrent coefficient, $I_{nor}$ is the rated current, and $m_{dc}$ is the maximum modulation index. *ω* is the angular frequency obtained from the PLL, and *U*_dc_ denotes the DC voltage, *L* denotes the equivalent impedance of the filter circuit. *u*_gd1_ and *u*_gq1_ are the d-axis and q-axis positive sequence reference voltage in the reference coordinate system, respectively. $\cos\varphi_{A,B,C}$ denotes the included angle between *V*_1_ and *V*_2_ of the corresponding phase.

By combining (10) and (11), the current reference $I_{ref}$ subject to the current-limiting constraint can be expressed as:

(12) $$I_{ref}=\min(I_{\max1},I_{\max2})$$

### 3.2. Directional Element Based on Control and Protection Coordination

Based on the above analysis, the fault direction can be inferred from the positive-sequence voltage–current phase relationship when the system impedance angle is 90°. In practical AC transmission systems, the impedance angle of overhead lines is generally within 70°~85°, and may approach 90° when the equivalent impedance is highly inductive. Therefore, 90° is adopted as a simplified reference angle for theoretical analysis and IBR fault-current phase control. This setting simplifies the IBR control strategy and avoids adjusting the control target according to the exact impedance angle of each line. Considering deviations in actual line and transformer impedance angles, the directional protection criterion is defined as follows:

(13) $$-90{}^{\circ}\leq \arg\frac{{\dot{U}}_{1}}{{\dot{I}}_{1}e^{j\varphi_{\mathrm{set}}}}\leq 90{}^{\circ}$$

where ${\dot{U}}_{1}$ denotes the positive-sequence voltage at the relay location, and ${\dot{I}}_{1}$ denotes the positive-sequence current defined as positive from the bus to the line. When the measured voltage falls below 30% of the rated voltage, ${\dot{U}}_{1}$ is replaced by the memorized voltage at the relay location. $\varphi_{\mathrm{set}}$ denotes the sensitivity angle of the protection. The system impedance angle, which is mainly determined by transmission lines and transformers, typically ranges from 70° to 90°. Considering the effect of transition resistance, the sensitivity angle is set to 80°.

In practical implementation, the proposed criterion uses the three-phase voltages and currents measured at the relay location as inputs. The fundamental-frequency phasors are first extracted by the relay phasor-calculation module, and the positive-sequence voltage ${\dot{U}}_{1}$ and current ${\dot{I}}_{1}$ are then obtained using the conventional symmetrical-component transformation. The current direction is defined as positive from the bus to the protected line. When the measured positive-sequence voltage is higher than the preset voltage threshold, the real-time ${\dot{U}}_{1}$ is used in (13). When the measured voltage falls below the threshold, ${\dot{U}}_{1}$ is replaced by the memorized positive-sequence voltage at the relay location to avoid the voltage dead-zone problem during close-in faults.

After ${\dot{U}}_{1}$ and ${\dot{I}}_{1}$ are determined, the phase relationship in (13) is calculated. If the compensated phase angle falls within the operating region, the fault is identified as being in the forward direction; otherwise, it is identified as a reverse fault. In the complete logic, the zero-sequence current is checked first. If zero-sequence current is present, the zero-sequence directional element is used. If no zero-sequence current is detected, the proposed positive-sequence criterion is applied to non-ground faults. In this way, the same calculation process and setting can be used at both line terminals.

**Figure 5:**
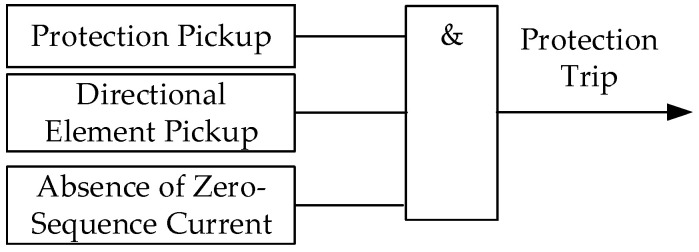
Decision logic of the proposed directional protection scheme for ground and non-ground faults.

According to the presence or absence of zero-sequence current at the relay location, the proposed criterion is coordinated with the zero-sequence directional protection to identify the fault direction. When zero-sequence current is present at the relay location, the zero-sequence directional element is used for fault-direction identification. When no zero-sequence current is detected, the same directional criterion is applied at both line terminals. Taking the protection at one terminal as an example, the decision logic of the proposed directional protection scheme is shown in Figure 5.

## 4. Performance Evaluation

### 4.1. Simulation Analysis

**Figure 6:**
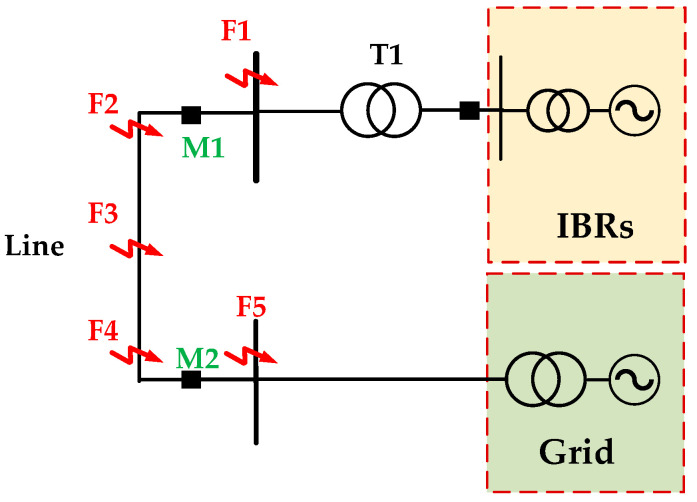
Diagram of power system with IBRs.

A PSCAD/EMTDC simulation model of an IBR-connected AC system is developed to verify the proposed directional protection method. The system parameters are listed in Table 1. The simulation model is established based on the parameters of an actual 220 kV regional power grid in China. As shown in Figure 6, M1 and M2 denote the line protection locations. Fault points F1~F5 are defined for testing: F1 and F5 correspond to reverse outlet faults with respect to the corresponding protection locations, F2 and F4 correspond to forward outlet faults, and F3 is located at the midpoint of the line. The performance of zero-sequence directional protection has already been verified [12]. Three-phase and phase-to-phase faults are simulated at F1~F5, and the verification results are summarized in Table 2 and Table 3.

Simulation results confirm that the proposed method reliably determines the fault direction in inverter-integrated AC systems, independent of fault location or type.

To further evaluate the robustness and adaptability of the proposed criterion, the influence of white noise, grid strength, and line X/R ratio is analyzed. The directional element at M1 is taken as the study object, and the fault at F3 is selected as a representative case.

To evaluate the influence of measurement noise on the proposed criterion, white noise is superimposed on the measured voltage and current signals. A low-pass filter with a cut-off frequency of 300 Hz is adopted before phasor extraction, and a 20 ms data window is used to calculate the fundamental-frequency phasors. The phase difference is then calculated and compared with the operating region of the proposed criterion. The results are shown in Table 4.

As shown in Table 4, the calculated positive-sequence voltage–current phase difference changes slightly after white noise is added to the measured signals. However, the phase difference still remains within the operating region for forward faults and outside the operating region for reverse faults. Therefore, the protection decision is unchanged under the tested noise condition. This indicates that, with the adopted low-pass filtering and 20 ms phasor-calculation window, the proposed criterion has acceptable robustness against measurement noise.

To further investigate the influence of grid-strength variation, the strength of the opposite-side AC system is changed by adjusting the equivalent source impedance. The protection at M1 is still taken as the study object, and the fault at F2 is selected as a representative forward fault. The corresponding protection decisions under different equivalent source impedance scaling factors are shown in Table 5.

As shown in Table 5, when the opposite-side grid strength varies within the tested range, the proposed criterion still identifies the fault direction correctly. This is because the proposed directional element is mainly based on the positive-sequence voltage–current phase relationship rather than the absolute magnitude of the fault current. Moreover, the angular operating region of the criterion provides a certain margin for practical variations in system strength. Therefore, moderate changes in the opposite-side grid strength do not change the basic directional decision of the proposed method.

To further examine the influence of line impedance characteristics, different line X/R ratios are considered. The variation in the X/R ratio changes the equivalent impedance angle and therefore affects the positive-sequence measured impedance angle used by the proposed criterion. The protection at M1 and the fault at F2 are selected as representative cases, and the corresponding results are shown in Table 6.

The results in Table 6 show that the proposed criterion remains effective under the tested X/R-ratio conditions. When the X/R ratio decreases, the equivalent impedance angle becomes smaller, and the calculated positive-sequence voltage–current phase difference shifts accordingly. However, because the operating region of the proposed criterion covers the practical impedance-angle range of AC transmission lines, the directional decision is not affected in the tested cases. If the line impedance angle deviates significantly from the normal transmission-line range, the sensitivity angle should be rechecked according to the actual line parameters.

### 4.2. Experimental Validation

**Figure 7:**
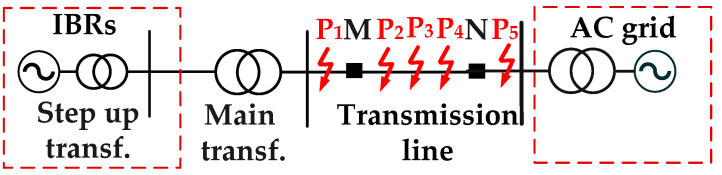
Diagram of tested power system.

**Figure 8:**
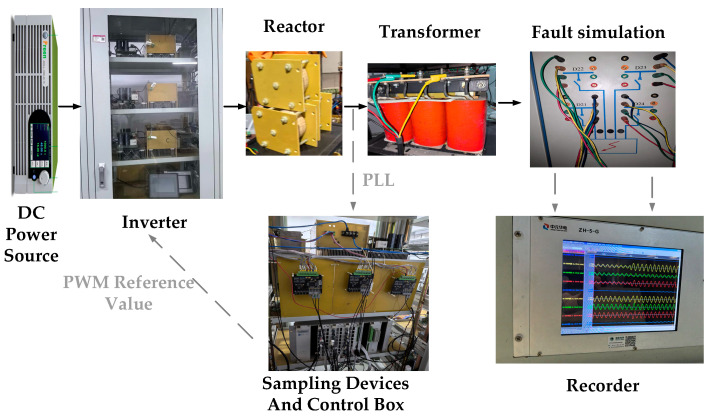
Schematic diagram of dynamic simulation experiment platform.

To further assess the engineering feasibility of the proposed method, a dynamic-model test system is built on the basis of the PSCAD/EMTDC scheme shown in Figure 7, and the corresponding experimental platform is presented in Figure 8. The test system emulates fault conditions on a 110 kV AC line connected to a 75 MW IBR. Fault points P1–P5 are arranged in the same manner as F1–F5 in the simulation study, so that the simulation and experiment can be directly compared.

To assess the suggested method, three-phase and phase-to-phase short-circuit faults are simulated at P1~P5, with the operation results summarized in Table 7 and Table 8. Dynamic simulation results confirm that the proposed method reliably determines the fault direction in inverter-integrated AC systems, independent of fault location or type.

**Figure 9:**
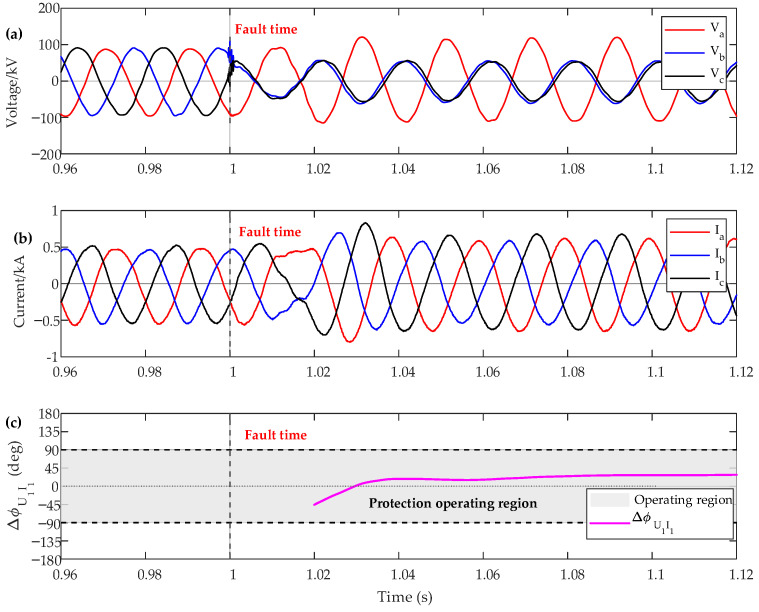
The experience result of the protection criteria of the IBR side under a phase-to-phase fault. (**a**) Voltage, (**b**) Current, (**c**) positive-sequence voltage–current phase difference.

**Figure 10:**
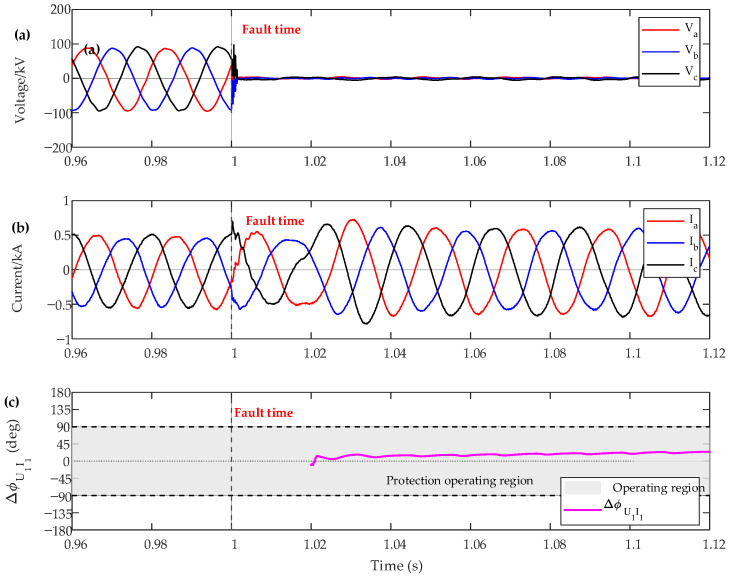
The experience result of the protection criteria of the IBR side under a three-phase fault. (**a**) Voltage, (**b**) Current, (**c**) positive-sequence voltage–current phase difference.

Figure 9 and Figure 10 illustrate the dynamic simulation test results and the corresponding protection decision results for a three-phase fault and a phase-to-phase fault at P2, respectively. It can be seen that the proposed method accurately identifies the fault location under both fault conditions. Moreover, no voltage dead zone is observed during near-zone faults, which further verifies the effectiveness of the proposed criterion.

**Figure 11:**
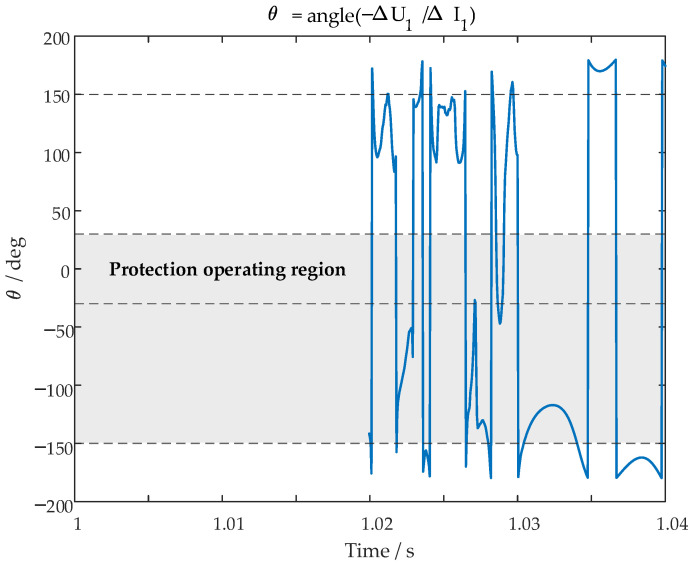
Operating result of the conventional incremental-quantity directional element under a three-phase fault at P2 with the IBR adopting a conventional FRT strategy.

When the IBR adopts a conventional FRT strategy, the operating result of a conventional incremental-quantity directional element is used for comparison. Taking the protection installed at the M side as an example, when a three-phase fault occurs at P2 on the line, the trajectory of the conventional incremental-quantity directional criterion is shown in Figure 11. After fault inception, the phase-synchronization relationship between the IBR PLL and the opposite-side AC system is disrupted by the IBR control response. As a result, the incremental-quantity directional criterion exhibits significant oscillations and cannot form a stable directional decision. Therefore, the conventional incremental-quantity directional element fails to reliably identify the fault direction under this condition.

## 5. Conclusions

This paper proposes a control–protection coordinated directional element for non-ground faults on AC lines connected to IBRs. The proposed method is developed from the positive-sequence voltage–current phase relationship at the relay location. By regulating the positive-sequence fault-current phase of the IBR, the fault characteristics required by directional protection are actively shaped, so that a stable and distinguishable directional feature can be established. On this basis, a unified angular criterion is formulated, which can be applied at both line terminals.

The PSCAD/EMTDC simulation verification and dynamic-model experimental validation demonstrate the effectiveness of the proposed method. For the tested phase-to-phase and three-phase faults at five fault locations and two relay locations, the proposed criterion achieves correct directional identification in the simulation and experimental cases, with no observed maloperation or failure to operate. The additional tests considering measurement noise and opposite-side grid-strength variation show that the protection decision remains unchanged under the tested disturbances. Compared with conventional positive-sequence directional elements, the proposed method improves the adaptability of non-ground fault-direction identification by actively shaping the positive-sequence voltage–current phase relationship through control–protection coordination.

The proposed method also has some limitations. It is mainly developed for grid-following IBR-connected AC line scenarios and depends on the availability of the IBR fault-current phase-control function. Its applicability to systems in which both sides are dominated by power-electronic sources, such as offshore wind power transmission systems, has not been fully investigated. In addition, when wind farms include other types of generators, such as doubly fed induction generators, the corresponding fault-current characteristics and the adaptability of the proposed criterion need to be further analyzed.

## Figures and Tables

**Table 1:** Parameters of the simulation system.

Description	Values
Capacity of IBRs	50 MW
Length of Line	20 km
Positive-sequence impedance of line	0.067 + j0.379 Ω/km
Zero-sequence impedance of line	0.345 + j1.003 Ω/km
Capacity of transformer T1	50 MVA
Positive-sequence leakage reactance of T1	0.1 p.u.
Connection type of T1	Yd11
Turns ratio of T1	220 kV/35 kV
Rated AC system voltage	220 kV
Short-circuit ratio of AC system	5

**Table 2:** PSCAD/EMTDC simulation verification results under phase-to-phase faults.

Fault Location	Relay Location	
	M1	M2
F1	R	F
F2	F	F
F3	F	F
F4	F	F
F5	F	R

F denotes forward direction; R denotes reverse direction.

**Table 3:** PSCAD/EMTDC simulation verification results under three-phase faults.

Fault Location	Relay Location	
	M1	M2
F1	R	F
F2	F	F
F3	F	F
F4	F	F
F5	F	R

F denotes forward direction; R denotes reverse direction.

**Table 4:** Influence of white noise on the proposed directional criterion.

Fault Type	SNR/dB	Decision
Phase-to-phase fault	0	F
	20	F
	30	F
Three-phase fault	0	F
	20	F
	30	F

F denotes forward direction.

**Table 5:** Influence of opposite-side grid strength on the proposed directional criterion.

Fault Type	Short-Circuit Ratio	Decision
Phase-to-phase fault	3	F
	5	F
	10	F
Three-phase fault	3	F
	5	F
	10	F

F denotes forward direction.

**Table 6:** Influence of line impedance on the proposed directional criterion.

Fault Type	Impedance Angle	X/R-Ratio	Decision
Phase-to-phase fault	70°	2.75	F
	80°	5.67	F
	85°	11.43	F
Three-phase fault	70°	2.75	F
	80°	5.67	F
	85°	11.43	F

F denotes forward direction.

**Table 7:** Dynamic-model results of the tested system (phase-to-phase fault).

Fault Location	Relay Location	
	M	N
P1	R	F
P2	F	F
P3	F	F
P4	F	F
P5	F	R

F denotes forward direction; R denotes reverse direction.

**Table 8:** Dynamic-model results of the tested system (three-phase fault).

Fault Location	Relay Location	
	M	N
P1	R	F
P2	F	F
P3	F	F
P4	F	F
P5	F	R

F denotes forward direction; R denotes reverse direction.

## Data Availability

The datasets generated and analyzed during the current study are available from the first author upon reasonable request.
